# Regional shape, global function and mechanics in right ventricular volume and pressure overload conditions: a three-dimensional echocardiography study

**DOI:** 10.1007/s10554-020-02117-8

**Published:** 2021-01-03

**Authors:** Jurate Bidviene, Denisa Muraru, Francesco Maffessanti, Egle Ereminiene, Attila Kovács, Bálint Lakatos, Jolanta-Justina Vaskelyte, Remigijus Zaliunas, Elena Surkova, Gianfranco Parati, Luigi P. Badano

**Affiliations:** 1 Department of Cardiology, Medical Academy, Lithuanian University of Health Sciences, Eiveniu str. 2, LT-50009 Kaunas, Lithuania; 2 Institute of Cardiology, Medical Academy, Lithuanian University of Health Sciences, Kaunas, Lithuania; 3 Department of Cardiological, Neural and Metabolic Sciences, Istituto Auxologico Italiano, IRCCS, Milan, Italy; 4 Department of Medicine and Surgery, University Milano-Bicocca, Milan, Italy; 5 Heart and Vascular Center, Semmelweis University, Budapest, Hungary; 6 Cardiac Division, Department of Echocardiography, Royal Brompton Hospital, London, UK; 7 Independent Researcher, Milan, Italy

**Keywords:** Right ventricular shape, Right ventricular function, Regional curvature, Tetralogy of Fallot, Pulmonary hypertension, 3D echocardiography

## Abstract

Our aim was to assess the regional right ventricular (RV) shape changes in pressure and volume overload conditions and their relations with RV function and mechanics. The end-diastolic and end-systolic RV endocardial surfaces were analyzed with three-dimensional echocardiography (3DE) in 33 patients with RV volume overload (rToF), 31 patients with RV pressure overload (PH), and 60 controls. The mean curvature of the RV inflow (RVIT) and outflow (RVOT) tracts, RV apex and body (both divided into free wall (FW) and septum) were measured. Zero curvature defined a flat surface, whereas positive or negative curvature indicated convexity or concavity, respectively. The longitudinal and radial RV wall motions were also obtained. rToF and PH patients had flatter FW (body and apex) and RVIT, more convex interventricular septum (body and apex) and RVOT than controls. rToF demonstrated a less bulging interventricular septum at end-systole than PH patients, resulting in a more convex shape of the RVFW (r = − 0.701, p < 0.0001), and worse RV longitudinal contraction (r = − 0.397, p = 0.02). PH patients showed flatter RVFW apex at end-systole compared to rToF (p < 0.01). In both groups, a flatter RVFW apex was associated with worse radial RV contraction (r = 0.362 in rToF, r = 0.482 in PH at end-diastole, and r = 0.555 in rToF, r = 0.379 in PH at end-systole, respectively). In PH group, the impairment of radial contraction was also related to flatter RVIT (r = 0.407) and more convex RVOT (r = − 0.525) at end-systole (p < 0.05). In conclusion, different loading conditions are associated to specific RV curvature changes, that are related to longitudinal and radial RV dysfunction.

**Electronic supplementary material:**

The online version of this article (10.1007/s10554-020-02117-8) contains supplementary material, which is available to authorized users.

Right ventricular (RV) remodeling in response to different loading conditions includes not only adaptations of size and function, but also changes of its shape [1–3], and the latter have been reported to be predictive of outcome in different clinical situations [4, 5]. So far, the main focus of the research about the RV shape has been the curvature of the interventricular septum [6]. Conversely, little is known about the remodeling of the different regions of the RV in response to chronic pressure or volume overload. Part of this lack of knowledge was related to the complex three-dimensional anatomy of the RV [7], its peculiar contraction pattern [8], and the difficulty to obtain a comprehensive imaging of the RV by two-dimensional echocardiography (2DE) [9]. Recent advancements in three-dimensional transthoracic echocardiography (3DE) allow to encompass the entire RV in a single pyramidal dataset and to perform a detailed quantitative analysis of its size, shape and function [10].

Using 3DE, it has been reported that the regional curvature of the RV outflow tract and septum was more convex in patients with pulmonary hypertension (PH) compared with healthy controls, and that those changes were associated with impairment of RV pump function [3]. However, little is still known about the impact of RV shape changes on RV function and mechanics in patients with repaired tetralogy of Fallot (rToF), in whom there is a progressive RV remodeling associated with volume overload induced by postoperative pulmonary regurgitation [11].

Accordingly, we hypothesized that in addition to changes in RV size and function, volume and pressure overload may also affect the regional RV wall curvatures, and that those changes may be associated with differences in RV function and mechanics. Consequently, the purpose of this study was to compare the RV remodeling occurring in both pressure and volume chronic overload conditions by 3DE, and to determine the relationships between regional differences in RV shape with changes of RV function and mechanics.

## Methods

### Patient population

From May 2016 to January 2017, we enrolled 64 consecutive patients with chronic volume or pressure overload of the RV. Thirty-three rToF patients (58% women, age 20 ± 8 years) who underwent total surgical correction in their childhood (surgery age 8 ± 11 months; a duration from the surgery to the time of study recruitment 18.6 ± 7.3 years), resulting in severe pulmonary regurgitation without significantly elevated RV systolic pressure constituted the chronic volume overload group.

Thirty-one patients with PH (77% women, age 57 ± 14 years) and no more than moderate tricuspid regurgitation formed the group with RV pressure overload.

Patients with either pulmonary stenosis or prosthetic pulmonary valve, greater than mild aortic or mitral valve disease, residual intracardiac shunts, atrial fibrillation, left ventricle ejection fraction (EF) < 50%, pacemaker, or poor quality of echocardiographic images were excluded from the study.

The control group included 60 age- and sex- matched healthy volunteers (30 subjects for rToF and PH group each) selected from the Padua “3D Echo Normal” database (inclusion/exclusion criteria described previously) [12, 13].

Written informed consent was obtained from all volunteers, and the study was approved by the local Ethics Committee (protocol 2380P approved on 06/10/2011).

### Echocardiography

Transthoracic echocardiography was performed using a Vivid E9 scanner (GE Vingmed, Horten, Norway) equipped with M5S and 4 V probes. Digital loops were stored and analyzed offline. All measurements were performed according to the ASE/EACVI guidelines [14–16].

The apical 4-chamber and short-axis views with color Doppler and continuous wave Doppler tracings were acquired for tricuspid and pulmonary regurgitation quantification [15, 16]. Peak velocity of tricuspid regurgitation signal and right atrial pressure (estimated from inferior vena cava size and respiratory collapsibility) were used to calculate systolic pulmonary artery pressure [14]. In addition, according to the guidelines [17], other echocardiographic signs suggesting PH were assessed.

Multi-beat (4 or 6) full-volume datasets of both the RV and the left ventricle were acquired for 3DE analysis during breath-hold from the apical approach. A multislice display was used during acquisition to ensure a complete inclusion of the RV in the dataset [12]. Image depth and sector width were optimized to achieve a temporal resolution higher than 20 volumes per second.

RV volumes and EF were measured using a commercially available software package (4D RV-Function 2, TomTec Imaging Systems, Unterschleissheim, Germany) by a single experienced investigator using a validated protocol [18]. Quantification of 3D left ventricular volumes and EF was performed using 4D Auto LVQ™ (GE Healthcare) [19].

### RV shape quantification

**Fig. 1:**
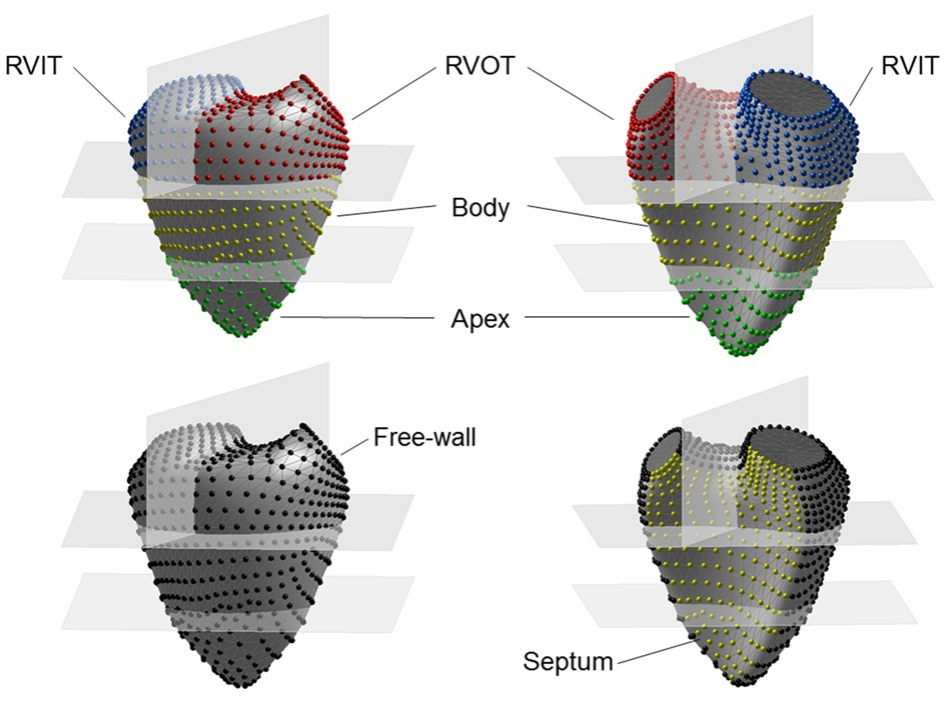
The 3D endocardial surface of the right ventricle (RV) was segmented in 4 subvolumes: inflow (RVIT) and outflow (RVOT) tracts, RV apex and body. The RV apex and body were further subdivided into free wall and septal components

The end-diastolic and end-systolic 3D RV surfaces were exported as meshes of connected points, and used as input into a custom-made software package to analyze the RV regional curvatures as previously described [20]. To identify the volumes of interest, a plane passing through the apex and both central points of the pulmonary and tricuspid valves was created. A second plane, perpendicular to the latter and passing through the RV apex and the mid-point between the two valves was used to discriminate between inflow and outflow subvolumes. Finally, the segment at the intersection between the two planes, going from the mid-point between the valve and the apex was subdivided into three equal parts to identify the 4 volumes of interest (Fig. 1): inflow (RVIT) and outflow (RVOT) tracts located in the upper third, RV body in the mid and RV apex (both of them divided into free wall (FW) and septum). The regional 3D curvature was obtained by averaging the values of all points within the region. In order to compensate for changes in local curvature secondary to changes in RV volume, the value of local curvature was normalized for the curvature of a sphere having the same instantaneous volume as the RV. Zero curvature defined a flat surface, whereas positive or negative curvature values indicated convexity or concavity, respectively. The more positive/negative the curvature, the more convex/concave the surface.

### Evaluation of 3D RV mechanics

**Fig. 2:**
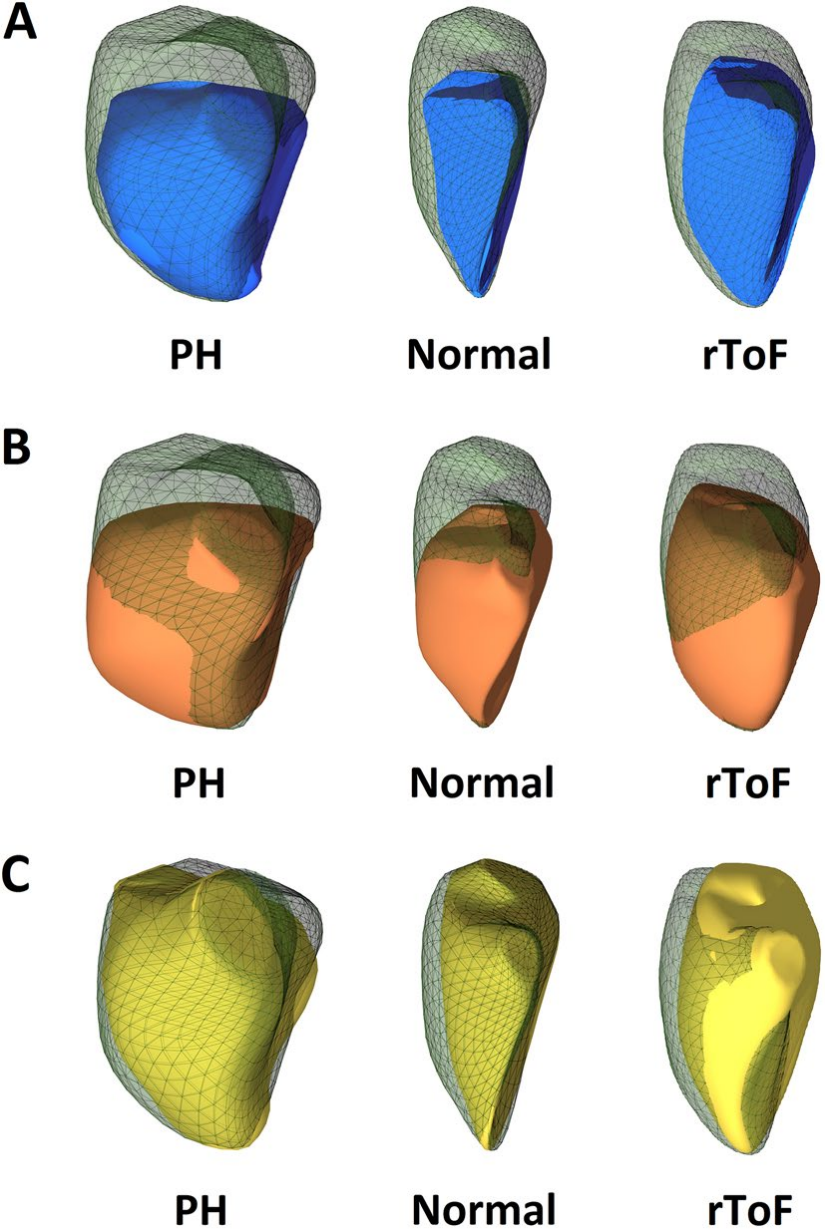
Global function (**a**) and decomposed longitudinal (**b**) and radial (**c**) wall motions of the right ventricle in pulmonary hypertension (PH) and repaired tetralogy of Fallot (rToF) patients, and a normal subject

The same exported 3D RV models were used as input to assess the longitudinal and radial RV wall motions and their relative contribution to global RVEF using the ReVISION method (Right VentrIcular Separate wall motIon quantificatiON; Argus Cognitive, Inc., Lebanon, New Hampshire, USA; www.revisionmethod.com) [21]. Briefly, the orientation of the exported 3D RV models was aligned using a standard, automated method to define the anatomically relevant, orthogonal axes (i.e., longitudinal or radial). Then, the wall motions of the 3D model were separated based on the movement of the model's each vertex point along these axes [22]. Movement in each direction can be selectively switched off to assess the contribution of those which remained enabled. This decomposition step transforms the original 3D model into new models, each corresponding to a decomposition type. For example, for quantifying the magnitude of longitudinal motion, we took into account the movement of the vertices along the RV vertical (longitudinal) axis only. Thus, volume changes attributable to either longitudinal or radial directions could be separately quantified and the corresponding EF values (i.e. longitudinal or radial) were calculated (Fig. 2). Finally, the relative contribution of the radial and longitudinal RV wall motion to global RV pump function was expressed by the ratio of the given directional EF to global RVEF.

### Statistical analysis

Statistical analysis was performed using SPSS version 24.0 for Windows (SPSS, Chicago, IL, USA). The qualitative variables were expressed in absolute numbers and percentages. The continuous variables were summarized using mean value ± standard deviation.

Kolmogorov–Smirnov test was used to assess distribution of variables. Normally distributed data were compared between rToF, PH and control groups with Student’s T test and in case of non-normality with Mann Whitney U test. Wilcoxon Signed Rank Test was used for the comparison of dependent variables. Pearson or Spearman correlation coefficient was used to assess correlations between RV shape and parameters of RV function.

Differences were considered statistically significant if p < 0.05.

## Results

### RV size and function in rToF and PH patients

In 29 (88%) rToF patients surgery was performed using transarterial approach, whereas the transventricular approach was used in the remaining 4 (12%) patients. Transannular patch was performed in 25 (76%) patients, whereas infundibulectomy was used in 4 (12%) patients. The surgical technique applied to the remaining 4 (12%) patients was unknown. Twenty-six (79%) rToF patients had right bundle branch block at the electrocardiogram, 13 (39%) rToF patients had moderate and 1 (3%) patient had severe tricuspid regurgitation. All rToF patients had severe pulmonary regurgitation.

The PH cohort included 21 (68%) patients with pulmonary arterial hypertension (PAH) (12 (39%) with idiopathic PAH, 7 (23%) with scleroderma associated PAH, and 2 (6%) with PAH associated to HIV infection), 8 (26%) with chronic thromboembolic PH, and 2 (6%) with chronic obstructive pulmonary disease. Twenty (65%) patients had mild and 11 (35%) had moderate tricuspid regurgitation.

Baseline demographics and clinical characteristics of the study cohort are summarized in Table 1. Left ventricular volumes were larger in rToF patients than in their controls despite the normalization to the body surface area. Left ventricular EF was lower in both rToF and PH patients than in their controls, but it was still within the normal range.

**Table 1:** Baseline clinical characteristics, left and right ventricular geometry and function of the study patients and controls

Characteristics, LV and RV parameters	rToF (N = 33)	Control-rToF (N = 30)	PH (N = 31)	Control-PH (N = 30)
Age (years)	20 ± 8	21 ± 6	57 ± 14^†^	57 ± 11
Women, n (%)	19 (58)	19 (63)	24 (77) ^†^	22 (73)
Body surface area (m^2^)	1.6 ± 0.23	1.8 ± 0.26*	1.8 ± 0.16^†^	1.7 ± 0.2
LV end-diastolic volume (ml/m^2^)	75 ± 13	65 ± 12*	63 ± 14^†^	57 ± 10
LV end-systolic volume (ml/m^2^)	32 ± 7	25 ± 6*	21 ± 11^†^	20 ± 4
LV EF (%)	58 ± 4	62 ± 4*	62 ± 7^†^	65 ± 3*
Pulmonary arterial systolic pressure (mmHg)	32 ± 7	21 ± 5*	58 ± 15^†^	21 ± 4*
RV free-wall S′ (cm/s)	10 ± 2	15 ± 2*	11 ± 3^†^	14 ± 2*
TAPSE (mm)	18 ± 3	25 ± 4*	21 ± 5^†^	26 ± 3*
RV fractional area change (%)	37 ± 5	49 ± 7*	26 ± 7^†^	51 ± 6*
Basal RV diameter (mm)	51 ± 10	37 ± 3*	50 ± 8	36 ± 4*
Mid-cavity RV diameter (mm)	47 ± 9	27 ± 3*	42 ± 8^†^	26 ± 5*
RV long-axis diameter (mm)	87 ± 10	69 ± 8*	79 ± 7^†^	66 ± 7*
RV end-diastolic volume (ml/m^2^)	133 ± 34	52 ± 18*	95 ± 22^†^	58 ± 13*
RV end-systolic volume (ml/m^2^)	74 ± 24	24 ± 10*	61 ± 20^†^	24 ± 6*
RV EF (%)	45 ± 6	55 ± 5*	36 ± 8^†^	59 ± 7*
Longitudinal RV EF (%)	17 ± 4	28 ± 4*	18 ± 5	25 ± 5*
Radial RV EF (%)	20 ± 5	25 ± 4*	13 ± 5^†^	27 ± 6*
Ratio of longitudinal to global RV EF	0.39 ± 0.09	0.52 ± 0.05*	0.51 ± 0.12^†^	0.46 ± 0.07
Ratio of radial to global RV EF	0.45 ± 0.08	0.46 ± 0.04	0.35 ± 0.11^†^	0.48 ± 0.07*

*EF* ejection fraction, *LV* left ventricular, *PH* pulmonary hypertension, *rToF* repaired tetralogy of Fallot, *RV* right ventricular, *TAPSE* tricuspid annular plane systolic excursion

^*^p < 0.05 comparing rToF and PH with controls; ^†^p < 0.05 comparing rToF and PH

As expected, rToF and PH patients showed significantly larger global and regional RV volumes both at end-diastole and end-systole, and lower RVEF than controls. rToF patients demonstrated larger RV mid-cavity and long-axis diameters, larger volumes (particularly at end-diastole) and higher RVEF compared with PH group (Tables 1 and 2).

**Table 2:** Global and regional right ventricular volumes and curvature parameters in study patients and controls measured at end-diastole and end-systole

Parameters	End-diastole				End-systole			
	rToF (n = 33)	Control-rToF (n = 30)	PH (n = 31)	Control-PH (n = 30)	rToF (n = 33)	Control-rToF (n = 30)	PH (n = 31)	Control-PH (n = 30)
RV volume (ml/m^2^)	130 ± 33	66 ± 16*	95 ± 23^†^	66 ± 14*	74 ± 24	30 ± 9*	62 ± 21 ^†^	31 ± 8*
RV apex (ml/m^2^)	21 ± 5	10 ± 3*	15 ± 4^†^	10 ± 2*	11 ± 4	4 ± 1*	9 ± 3 ^†^	4 ± 1*
RV body (ml/m^2^)	50 ± 13	25 ± 6*	36 ± 9^†^	25 ± 5*	28 ± 9	12 ± 4*	24 ± 8 ^†^	12 ± 3*
RVIT (ml/m^2^)	36 ± 10	18 ± 4*	27 ± 6^†^	18 ± 4*	21 ± 7	8 ± 3*	17 ± 6 ^†^	9 ± 2*
RVOT (ml/m^2^)	24 ± 6	12 ± 3*	18 ± 4^†^	12 ± 3*	14 ± 5	6 ± 2*	12 ± 4	6 ± 1*
Curvature (indexed to volume)								
RVIT	1.14 ± 0.1	1.23 ± 0.1*	1.19 ± 0.1	1.32 ± 0.3*	1.19 ± 0.1	1.13 ± 0.2	1.16 ± 0.1	1.25 ± 0.1*
RVOT	1.52 ± 0.1	1.44 ± 0.1*	1.49 ± 0.1	1.40 ± 0.1*	1.44 ± 0.1	1.28 ± 0.1*	1.43 ± 0.1	1.31 ± 0.1*
RV free-wall	1.36 ± 0.1	1.43 ± 0.1*	1.36 ± 0.1	1.42 ± 0.1*	1.37 ± 0.1	1.34 ± 0.1	1.29 ± 0.1^†^	1.35 ± 0.1*
Free-wall RV body	1.08 ± 0.1	1.20 ± 0.1*	1.09 ± 0.1	1.16 ± 0.1*	1.06 ± 0.1	1.16 ± 0.1*	1.01 ± 0.1^†^	1.09 ± 0.1*
Free-wall RV apex	1.95 ± 0.2	2.40 ± 0.2*	2.02 ± 0.3	2.34 ± 0.2*	2.05 ± 0.3	2.34 ± 0.2*	1.85 ± 0.2^†^	2.29 ± 0.2*
Interventricular septum	0.68 ± 0.1	0.60 ± 0.1*	0.69 ± 0.1	0.59 ± 0.1	0.66 ± 0.1	0.55 ± 0.1*	0.76 ± 0.1^†^	0.58 ± 0.1*
Septal RV body	0.40 ± 0.1	0.14 ± 0.1*	0.32 ± 0.1 ^†^	0.18 ± 0.1*	0.37 ± 0.2	0.15 ± 0.1*	0.46 ± 0.1^†^	0.2 ± 0.1*
Septal RV apex	1.01 ± 0.3	0.54 ± 0.3*	0.98 ± 0.3	0.38 ± 0.2*	1.14 ± 0.4	0.62 ± 0.4*	1.19 ± 0.5	0.46 ± 0.3*

*PH* pulmonary hypertension, *rToF* repaired tetralogy of Fallot, *RV* right ventricle, *RVIT* right ventricular inflow tract, *RVOT* right ventricular outflow tract

^*^p < 0.05 comparing rToF and PH with controls; ^†^p < 0.05 comparing rToF and PH

### Regional RV shape in rToF and PH patients

**Fig. 3:**
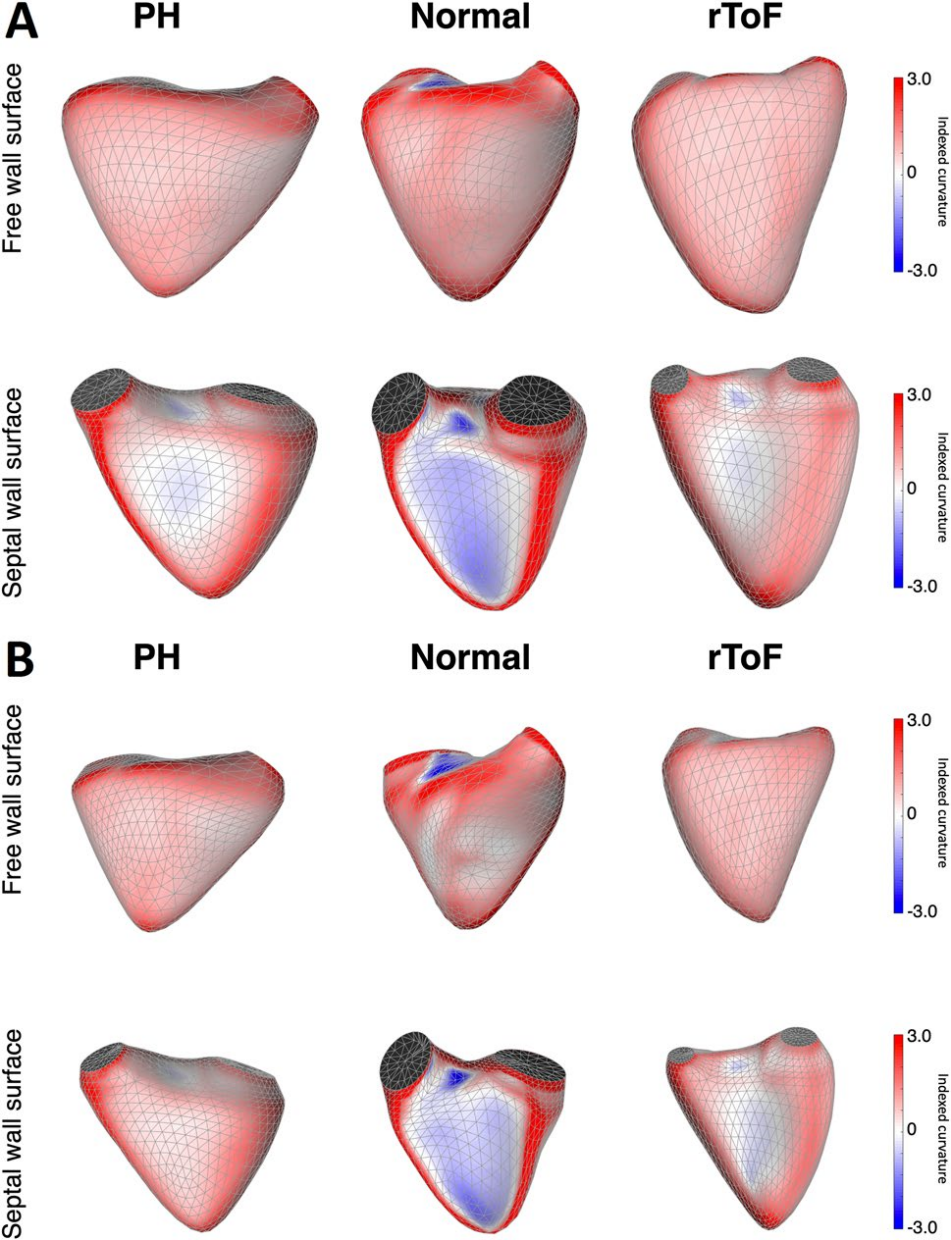
Colour-coded map of mean 3D curvature values obtained in repaired tetralogy of Fallot (rToF) and pulmonary hypertension (PH) patients, and a normal subject at end-diastole (**a**) and end-systole (**b**). Blue denotes more concave surface and red denotes more convex. In rToF and PH patients, the right ventricular free wall is flatter, and the interventricular septum is more convex compared to normal subject throughout the cardiac cycle. In rToF, the interventricular septum curvature is more convex at end-diastole (**a**) comparing with PH patients, but this relationship is inverted at end-systole (**b**)

**Fig. 4:**
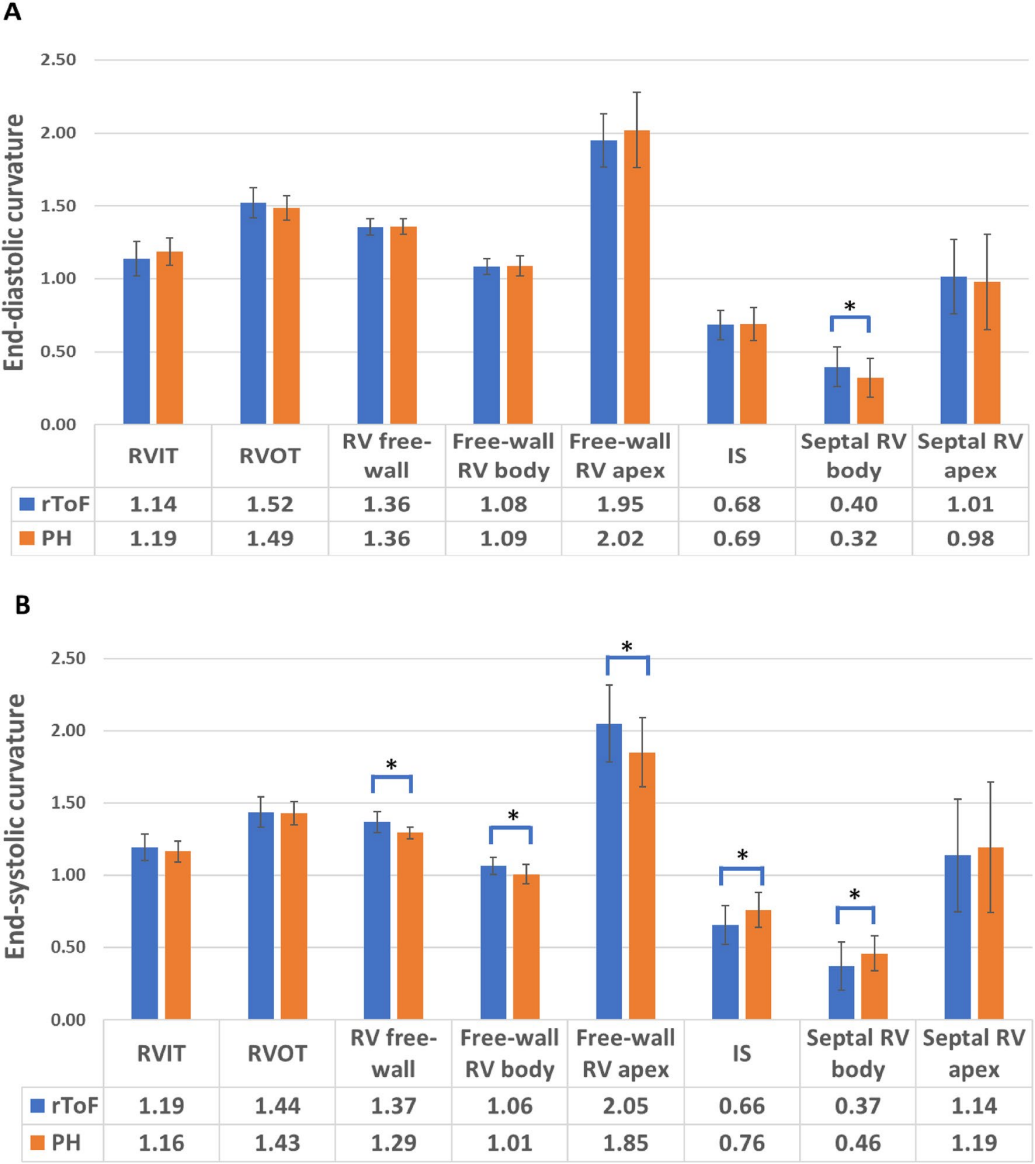
Bar graphs comparing end-diastolic (**a**) and end-systolic (**b**) regional curvature values for the patients with repaired tetralogy of Fallot (rToF, blue bars) and patients with pulmonary hypertension (PH, orange bars). *IS* interventricular septum, *RV* right ventricular, *RVIT* right ventricular inflow tract, *RVOT* right ventricular outflow tract. *p < 0.05

Both rToF and PH patients demonstrated less convex RVFW (both body and apical segments) and RVIT, more convex interventricular septum (both body and apical segments) and RVOT than controls (Table 2, supplemental video 1–6, https://figshare.com/s/14c22bd00065d3de9ff3). The RV septal body curvature was more convex in rToF than in PH patients at end-diastole, but this relationship was inverted at end-systole (Table 2, Fig. 3, 4, Video 4 and 6). In rToF patients, a less bulging interventricular septum at end-systole resulted in a more convex shape of the whole RVFW (r = − 0.701, p < 0.0001) compared to PH group. Septal apex curvature was similar in both groups of patients (Table 2, Fig. 4).

In both groups, RVOT became significantly less convex at end-systole than at end-diastole (p < 0.0001). RVIT convexity increased significantly from end-diastole to end-systole in rToF patients (p < 0.0001), while it did not change throughout the cardiac cycle in PH patients (p > 0.05) (Table 2).

Dynamic RV shape changes (i.e., differences between end-diastole and end-systole) were more pronounced in PH group (Table 3). Respectively, in PH patients the septal body curvature significantly increased from end-diastole to end-systole, whereas in rToF patients its convexity tended to decrease in systole (Fig. 3). Similarly, although the RVFW body also progressed to a less convex shape in end-systole in rToF (delta rToF = 0.02 ± 0.06), the curvature difference between end-diastole and end-systole was less pronounced compared with PH group (delta PH = 0.08 ± 0.06, p < 0.0001). Moreover, in rToF the RVFW body segment became less convex from end-diastole to end-systole, while the FW apex changed to a more convex shape. In PH both RVFW components (apex and body) became less convex during the cardiac cycle (Table 2 and 3). However, there was a drop in dynamic changes of RVFW body curvature throughout the cardiac cycle in rToF patients (p = 0.048) comparing with PH (p < 0.0001).

**Table 3:** Dynamic RV shape changes from end-diastole to end-systole in rToF and PH patients

Curvature (indexed to volume)	Change in curvature (Delta) from end-diastole to end-systole	
	rToF (n = 33)	PH (n = 31)
RVIT	− 0.05 ± 0.06	0.02 ± 0.08*
RVOT	0.08 ± 0.09	0.06 ± 0.06
RV free wall	− 0.01 ± 0.05	0.06 ± 0.04*
Free-wall RV body	0.02 ± 0.06	0.08 ± 0.06*
Free-wall RV apex	− 0.10 ± 0.22	0.17 ± 0.15*
Interventricular septum	0.03 ± 0.09	− 0.07 ± 0.09*
Septal RV body	0.02 ± 0.11	− 0.14 ± 0.10*
Septal RV apex	− 0.12 ± 0.26	− 0.21 ± 0.26

*PH* pulmonary hypertension, *rToF* repaired tetralogy of Fallot, *RV* right ventricle, *RVIT* right ventricular inflow tract, *RVOT* right ventricular outflow tract. *p < 0.0001

### The relation between regional RV shape and function in rToF and PH patients

In both rToF and PH patients, a more convex RVOT at end-systole was associated to lower RV fractional area change (r = − 0.423, p = 0.03 in rToF and r = − 0.484, p = 0.007 in PH) and EF (r = − 0.347, p = 0.048 and r = − 0.424, p = 0.02, respectively), and this association was stronger in PH. Moreover, in PH patients more convex RVOT at end-systole was associated with lower TAPSE (r = − 0.455, p = 0.012) and S’ velocity of the RVFW (r = − 0.551, p = 0.002).

In rToF patients, regional curvature values for the whole RV interventricular septum at end-systole demonstrated moderate negative correlation with RV fractional area change (r = − 0.386, p = 0.03) and a tendency to negative correlation with RVEF (p = 0.094). In PH patients, the convexity of the interventricular septum at end-diastole showed a tendency to negatively correlate with TAPSE (p = 0.07) and RVEF (p = 0.059).

In PH patients, RVIT curvature at end-systole correlated positively with RV fractional area change (r = 0.387, p = 0.035) and EF (r = 0.386, p = 0.035).

### The relation between regional RV shape and mechanics in rToF and PH patients

The relative contribution of the longitudinal wall motion to global RVEF was significantly lower in rToF compared with their control group. Whereas, in PH patients it was the relative contribution of the radial component of RV wall motions to be more impaired in comparison to their controls (Table 1, Fig. 2).

In both groups, the RVFW apex curvature at end-diastole (r = 0.362, p = 0.038 in rToF and r = 0.482, p = 0.007 in PH) and at end-systole (r = 0.555, p = 0.001 in rToF and r = 0.379, p = 0.039 in PH, respectively) demonstrated a positive correlation with the radial RVEF. In addition, in rToF patients, the worse radial RV contraction was associated with less convex curvature of the whole RVFW (r = 0.420, p = 0.015) and the more convex septal body curvature (r = − 0.401, p = 0.02) at end-systole. In PH patients, the impairment of radial RVEF was also related to less convex RVIT (r = 0.407, p = 0.026) and more convex RVOT (r = − 0.525, p = 0.003) at end-systole.

In rToF patients, the deterioration in longitudinal RVEF was associated with more convex curvature of the whole RVFW at end-systole (r = − 0.397, p = 0.02) and RVFW apex at end-diastole (r = − 0.363, p = 0.038). In PH patients, the longitudinal RVEF demonstrated negative correlation with the whole RV septum curvature in diastole (r = − 0.417, p = 0.022).

## Discussion

As far as we know, this is the first study that used 3DE to assess regional RV shape differences in patients with distinct loading conditions of the RV and to study their relationships with RV function and mechanics. We demonstrated that: *i*. both rToF and PH patients had larger RV volumes and lower EF, flatter (less convex) RVFW and RVIT, more convex septum and RVOT than their age and sex-matched controls; *ii*. in rToF patients the RVFW body segment became flatter, while the FW apex changed to a more convex shape, whereas in PH patients, both RVFW components (apex and body) became flatter from end-diastole to end-systole; *iii*. A more convex RVOT at end-systole was associated with impaired RV systolic function in rToF and PH patients; *iv*. compared with PH patients, rToF showed a less convex RV septal body curvature at end-systole that was associated with a more convex shape of the RVFW, and worse longitudinal RVEF; *v.* both in rToF and PH patients, a flatter RVFW apex curvature was associated with worse radial RVEF, meanwhile in PH group, the impairment of radial RV contraction was also related to flatter RVIT and more convex RVOT at end-systole.

### The importance of regional RV shape assessment in rToF patients

Early surgical ToF repair often leaves patients with severe residual pulmonary regurgitation, which causes progressive RV dilation and late complications [23, 24]. Timely pulmonary valve replacement performed before the occurrence of irreversible RV dysfunction, might prevent these complications [25]. According to our data, in rToF patients, the chronic volume overload dilates the RV more than the pressure overload of the PH patients and determines a more spherical RV shape in the former. However, since RV dilation results in significant regional differences [2, 26–28], traditional parameters of RV geometry and function may not provide an accurate quantitative description of the RV remodeling occurring in rToF patients. Conversely, the study of the regional remodeling of the RV allows to understand the way the RV adapts to the volume and the pressure overload in rToF and PH.

When RV dilates, contractile function increases according to the Frank-Starling law, which for some time effectively compensates for altered hemodynamic conditions. However, the adaptive cardiac response to pathological loading conditions is time dependent and long-term chronic RV volume overload due to severe pulmonary regurgitation leads to stretching of myofibers with subsequent distorted meshwork, more spherical architecture, RV dilatation and decreased contractility [29, 30]. These structural changes might modify the contraction pattern to a greater radial contribution [30]. Accordingly, we demonstrated that in rToF patients with RV volume overload the longitudinal component of RV wall motion is impaired more than the radial one. These findings could explain the phenomenon clinical practice why some rToF patients with reduced TAPSE still have maintained RVEF. However, relevant literature also suggests another possible explanation for such changes in rToF: Sanchez-Quintana et al. demonstrated that in patients with ToF the longitudinal layer of RV myocardium has a more oblique orientation, and also the middle circumferential layer, which is not dominant in physiological conditions, was found to be more significant in these patients [31]. Thus, it is of scientific interest whether these changes in myofiber architecture are inherited or just consequences of chronic volume overload of the chamber.

Moreover, when RV preload increases, wall stress is maintained by dilation of the chamber. Previously, it was demonstrated that regional curvature differences are related to regional wall stress [32]: decreased curvature is associated with increased wall stress. Similar to previous findings [26], we demonstrated that rToF patients had significantly flatter RVFW (body and apex) than controls. In addition, we observed that a more convex RVFW in systole was associated with worse longitudinal and better radial RVEF in rToF group.

The apical trabecular component of the RV yields the major adaptive response in rToF patients with volume overload [27]. Our results also showed that the RV apex became larger and flatter in comparison to controls, while becoming more convex than the other RV segments. These findings are concordant with previous reports [2, 26]. In addition, we found that the flatter RVFW apex was associated with the impairment of the radial RVEF. Moreover, we demonstrated a more convex RVFW apical shape during systole in rToF than in PH patients, and these changes could be related to better radial RVEF in rToF patients. Conversely, a more convex FW apex curvature in diastole was associated with lower longitudinal RVEF in rToF patients.

The results of our study also confirmed the observations that rToF patients with severe pulmonary regurgitation had significantly more convex RVOT compared to controls throughout the cardiac cycle [33]. However, several previous studies hypothesized that the changes in RVOT curvature also may be associated with postsurgical scarring [27, 34], so larger future studies are needed to clarify the relationship between the RVOT curvature and RV volume overload. In addition, we found that a more convex RVOT was associated with worse RV systolic function. Our observations were consistent with the previous findings [35] that RVOT regional abnormalities adversely affect global RV function and exercise capacity after ToF repair. However, the RVOT assessment remains difficult both by cardiac magnetic resonance imaging and 2DE [36], thus 3DE could play an important role in the follow-up of the rToF patients.

The abnormal motion of the interventricular septum is a well known echocardiographic feature of both RV volume and pressure overload [37, 38], and an eccentricity index may help to discriminate between RV volume overload and pressure overload states [1]. However, 2D techniques can only measure the component of curvature in the direction of the imaging plane and slightly off-axis images can result in artefactually flattened septum and an incorrect calculation of the eccentricity index. 3DE includes the entire interventricular septum and therefore is more accurate in describing septal curvature changes during the cardiac cycle [39]. We demonstrated that rToF patients had more convex interventricular septum than controls during cardiac cycle. Moreover, we showed that the volume overload pushed less the septal RV body at end-systole than pressure overload and this caused a more convex shape of the RVFW at end-systole to accommodate "large" RV volume.

### The importance of regional RV shape assessment in PH patients

In pressure overload, the RV hypertrophy has been described as the initial adaptive response to increase contractility [40]. Several investigators have suggested that the myocardial hypertrophy that develops in pressure overload may be a useful adaptive process in order to normalize myocardial stress [41, 42]. However, with the progression of the disease, RV wall stress increases because RV wall thickness does not increase proportionally [43]. As we described above, mechanical wall stress is inversely proportional to local RV curvature [32]. We demonstrated that in PH multiple RV regions (excluding RVOT and septum) showed decreased local curvature during diastole comparing with controls, indicating increased wall stress in these regions. Thus, quantitative evaluation of RV shape not only allows better understanding of RV remodeling in PH, but also indirectly reflects the effects of pressure overload on mechanical stress.

Moreover, in patients with RV pressure overload, the RV acquire a unique shape compared to normals characterized by apical bulging (assessed by measuring the angle between the apex FW and the septum in the apical four-chamber view) and overall rounding of the RV apex [44]. Recently, these findings were confirmed using 3DE [3]. We showed that in PH patients the RV apex had flatter FW and was more convex at the septal part when compared with controls. Differently from rToF patients and controls, the apical FW segment of the RV became significantly flatter from end-diastole to end-systole. In addition, we demonstrated that in PH radial component of RV wall motion was affected more than its longitudinal shortening and a flatter apical RVFW segment shape was associated with a worse radial RVEF in these patients.

It was demonstrated, that in keeping with the “bellows-like” action of normal RV contraction, the FW body segment became flatter, while the apex FW changed to a more convex surface transitioning from end-diastole to end-systole [13]. In contrast, in PH, both RVFW segments (apical and body) remained equally convex throughout the cardiac cycle [3]. According to our results in PH patients both RVFW segments become less convex from end-diastole to end-systole. However, because of the relatively small number of healthy volunteers, we were not able to demonstrate the previously described ‘‘bellows-like “ action of normal RV contraction in controls, too [13]. Similar to Addetia et al. [3] we found that RVOT and the interventricular septum became more convex, the RVIT and the RVFW were flatter in PH than in their control group. Furthermore, we demonstrated that a more convex RVOT was associated with worse RV systolic function and decreased radial RVEF in this group of patients.

According to our results, a flatter RVIT in PH was associated with the impairment of the RV global systolic function and worse radial RVEF. Addetia et al. did not show any meaningful correlations of RVIT curvature with 2D and Doppler parameters of the right heart [3]. However, they found that RVIT curvature was a more robust predictor of death than RVEF, RV volumes, or other regional curvature indices, suggesting that patients who die have smaller RVIT curvature values and therefore more flat RV inflow regions.

Flattened septum and septal bowing towards left have been demonstrated as independent predictors of adverse events in PH and pulmonary embolism [4], and that regional 3D curvature values for the septum correlated inversely with the RV longitudinal function parameters [3]. We also showed that interventricular septum remains convex (i.e. bulging into the left ventricle) throughout the cardiac cycle, especially in systole, in PH patients and that a more convex interventricular septum at end-diastole was associated with worse longitudinal RVEF.

### Limitations

RV shape analysis using this custom software relies on accurately generated 3D endocardial surface model and therefore, it is dependent on the quality of the 3DE datasets. 3D analysis of regional endocardial curvature is fully automated once the endocardial surface is defined, and therefore it is 100% reproducible. In turns, the results could be affected by the variability of 3D RV endocardial reconstruction that was performed using a commercially available software. This issue has been already addressed in another publication where the methodology proved fair to excellent reproducibility in terms of intraclass coefficient of variation [13]. Moreover, in PH and rToF patients, the acquisition of good-quality 3D datasets could be affected by the size of the RV and the difficulty to accommodate large RV within the pyramidal dataset. However, experience in 3D acquisition and analysis might minimize this limitation.

The diagnosis of PH was confirmed by right heart catheterization. However, for many patients the right heart catheterization was done at the beginning of the follow-up and not all results were available, so we decided to use echocardiography to measure systolic pulmonary artery pressure.

Finally, the absence of a true reference method to test the accuracy of our measurements, could be considered as a limitation of this study and only prospective outcome studies can assess the clinical value of our approach.

## Conclusions

This is the first study to quantitatively define the differences of regional RV shape changes in rToF and PH patients and their relations with RV global function and mechanics using 3DE.

According to our findings, the RV in volume and pressure overload can be differentiated from normal RV using regional curvature–based analysis. Thus, since RV dilation results in regional inhomogeneity of shape, it is obvious that traditional parameters of RV geometry and function are not sufficient to assess the RV remodeling process occurring in rToF and PH. Moreover, our results suggest that RV remodeling in distinct loading conditions is significantly related to RV function and mechanics. This is the first study to show that changes in the shape of different RV parts affect RV mechanics. It is well known that some RV segments, such as RVOT, are very difficult to assess using standard 2DE. Thus, these findings highlight the importance of 3DE in more precise assessment of RV pump function in rToF and PH patients.

Consequently, quantitative analysis of regional curvature, as described in this study, could potentially be used to characterize the dynamic behaviour of the RV in distinct conditions and diseases, providing additive information which is not available using conventional parameters or even 3D volumes and EF. Moreover, ability to assess RV regional remodeling in different conditions and diseases could provide insights into pathological mechanisms, resulting in a useful support tool for disease evaluation and therapy planning.

## Electronic supplementary material

Below is the link to the electronic supplementary material. Right ventricular 3D curvature changes during cardiac cycle in a normal subject (**video 1 and 2**), pulmonary hypertension (PH) (**video 4 and 5**) and repaired tetralogy of Fallot (rToF) (**video 5 and 6**) patients. Blue denotes more concave surface and red denotes more convex. In rToF and PH patients, the right ventricular free wall is flatter, and the interventricular septum is more convex compared to normal subject throughout the cardiac cycle. In PH patients the septal body curvature significantly increases from end-diastole to end-systole, whereas in rToF patients its convexity decreases in systole.

Electronic supplementary material 1 (MOV 432 kb)

Electronic supplementary material 2 (MOV 438 kb)

Electronic supplementary material 3 (MOV 412 kb)

Electronic supplementary material 4 (MOV 436 kb)

Electronic supplementary material 5 (MOV 375 kb)

Electronic supplementary material 6 (MOV 398 kb)

## Abbreviations

2DE: Two-dimensional echocardiography
3DE: Three-dimensional echocardiography
EF: Ejection fraction
FW: Free wall
PH: Pulmonary hypertension
rToF: Repaired Tetralogy of Fallot
RV: Right ventricle/ventricular
RVIT: Right ventricular inflow tract
RVOT: Right ventricular outflow tract
TAPSE: Tricuspid annular plane systolic excursion
